# Olive Pomace-Derived Carbon Materials—Effect of Carbonization Pressure under CO_2_ Atmosphere

**DOI:** 10.3390/ma12182872

**Published:** 2019-09-05

**Authors:** Natalia Howaniec

**Affiliations:** Department of Energy Saving and Air Protection, Central Mining Institute, Pl. Gwarkow 1, 40-166 Katowice, Poland; n.howaniec@gig.eu; Tel.: +48-32-259-2219

**Keywords:** olive pomace, porous structure, carbon dioxide, pressure, waste valorization

## Abstract

The valorization of waste and by-products from various industrial activities is a must in our world of depleting natural resources and increasing volume of environmentally negative waste materials. The economic utilization of solid biowaste involves predominantly its use as a carbon-neutral energy resource or a precursor of porous carbon materials, with a potential application range including sorption processes, energy storage, and electric engineering. With the considerable number of lignocellulosic residues tested and applied as the most suitable porous material precursors, such as woods, shells, stones, peels, husks, and stalks of various crop plants, there is still space and need for further developments in the valorization of high amounts of other types of biowaste. Here, the olive pomace was considered because of both the vast volume and the environmentally undesired (when stored) phytotoxic effect of its components. While the literature on chemical (acidic and alkali treatment) and physical activation (temperature, carbon dioxide, and/or steam) of various biowaste precursors is considerable, the effects of pressure in the carbonization step are reported rarely, although the results observed are promising. The same applies to reports on the application of olive pomace for porous materials production, which indicate that olive pomace currently seems to be underestimated as a carbon materials precursor. In the study presented, the combined effects of pressure (0.1–3 MPa), temperature (800 °C), and carbon dioxide atmosphere in the carbonization of olive pomace were assessed on the basis of qualitative and quantitative data on micro- and mesoporosity of the carbon materials produced. The results showed the positive effect of increasing the process pressure on the development of a porous structure, and particularly, on the development of supermicropores and ultramicropores under the carbonization conditions applied. Carbon material with the most developed porous structure and the highest share of micropores was obtained under the maximum pressure tested.

## 1. Introduction

In the world of depleting natural resources, waste biomass is considered to be a valuable, carbon-neutral resource for various industrial applications, covering predominantly energy systems where waste biomass is applied in the production of energy and hydrogen in gasification and co-gasification [1,2,3,4] or methane-rich gas (biogas) in anaerobic digestion [5]. The economic utilization of waste in energy production is placed alongside the incorporation of carbon dioxide and excess industrial heat in the low carbon-footprint valorization cycles of sustainable economic systems [6,7,8].

Another important method of waste biomass valorization is the production of carbon materials with enhanced sorption properties for environmental applications, including removal of contaminants from gaseous and liquid phases. In this case, lignocellulosic residues are most often applied as the most suitable precursors for the production of the so-called activated carbons and include woods, shells, stones, peels, husks, and stalks of various crop plants [9]. Among them, olive tree residues are often considered, since the market for olive oil has doubled over the last 20 years, reaching an olive oil production of over three million tons in 2018 [10]; therefore, the volume of the related biowaste is large and still growing [11]. Olive pomace is a lignocellulosic material containing about 5–10% of proteins and 4–15% of oil, and it is the main waste generated in olive oil production—about 40% by weight of raw olive processed [12]. It also contains high amounts of phenolics, mainly hydroxytyrosol and its derivatives, with environmentally undesired phytotoxic effects [13]. Various research studies examining the application of olive stones [14,15,16,17,18] and, less extensively, of olive tree pruning residues [19,20] or mill waste [21,22,23,24,25,26] as activated carbon precursors are available in the literature, reporting their physical and chemical treatment effects and applicability in CO_2_, NO_2_, CH_4_, and gasoline vapors capture [27,28,29,30,31,32], as well as for dyes and heavy metals removal from aqueous solutions [21,24,25,26]. The activation of a raw precursor usually involves chemical treatment with acidic and/or alkali agents, like H_2_SO_4_ [31], H_3_PO_4_ [19,21,23,30,31], or KOH [20,23,31], followed by carbonization under inert gas atmosphere at an increased temperature, typically of 350–800 °C. In some works, carbon dioxide and steam activation of carbonized olive stones are reported [15,16,17,18,28,32,33,34]. Limited data are available, however, on the use of pressure as an activation agent in tailoring the porous structure of carbon materials, and these works are mainly related to bituminous coal or lignite as precursors [34,35,36,37,38], with a few works devoted to biomass treatment under elevated pressure and temperature for the development of carbon materials of increased porosity [18,39,40].

The potential of olive pomace in environment-friendly valorization cycles, together with the scarce data on the role of carbonization pressure alone and in combination with other parameters in tailoring the porous structure properties of waste-derived carbon materials were the main reasons prompting the study presented in his paper. Experimental data on olive pomace-derived carbon materials preparation are limited and practically missing when activation with pressure and carbon dioxide under elevated temperature is considered. These are, therefore, the main rationales of the study presented. Olive pomace was treated under inert gas atmosphere at 800 °C and under the pressure of 1–3 MPa and then activated with carbon dioxide for 10 min. A wide range of porous structure parameters of the resulting chars were determined, providing qualitative and quantitative data on their micro- and mesoporosity, which are important for their potential application as prospective porous materials.

## 2. Materials and Methods

The proximate and ultimate analyses of olive pomace as a carbon material precursor were performed in compliance with the relevant standards, and the results are given in Table 1.

**Figure 1:**
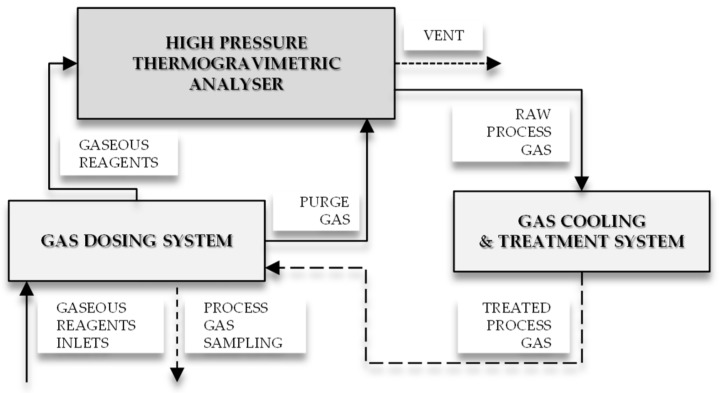
Schematic diagram of the experimental setup for biomass carbonization.

An amount of 1 g of olive pomace biomass was placed in a crucible of a high-pressure thermogravimetric analyzer (Rubotherm GmbH, Bochum, Germany), heated to 800 °C at the rate of 20°C/min in an inert gas atmosphere (Ar) and pressurized to 1, 2, or 3 MPa (see Figure 1). Next, under the final process conditions of pressure and temperature, biomass char was treated with carbon dioxide supplied to the reactor at a flow rate of 100 mL/min for 10 min. Tests under atmospheric pressure were also performed for comparison.

The analysis of the porous structure parameters of the resulting carbon materials was based on the nitrogen and carbon dioxide isotherms. Prior to the measurements performed with the use of the gas sorption analyzer Autosorb iQ (Quantachrome Instruments, Boynton Beach, FL, USA), the samples were outgassed at 120 °C under vacuum conditions overnight. The pore size distribution of the micro- and mesoporous materials was analyzed with the use of the density functional theory (DFT) [41,42], and the specific surface area was quantified with the application of the multi-point Brunauer-Emmett-Teller (BET) method [43] on the basis of the nitrogen sorption isotherm (−196 °C). The total pore volume was considered to be the volume at the relative pressure of 0.99. The carbon dioxide isotherm at 0 °C was the basis for the determination of the narrow micropores’ area and volume by the Monte Carlo method [44]. The scanning electron microscopy (SEM) technique was employed to examine the textural properties of the resulting chars with the application of the SU-3500N microscope (Hitachi High-technologies Corporation, Tokyo, Japan).

## 3. Results and Discussion

**Figure 2:**
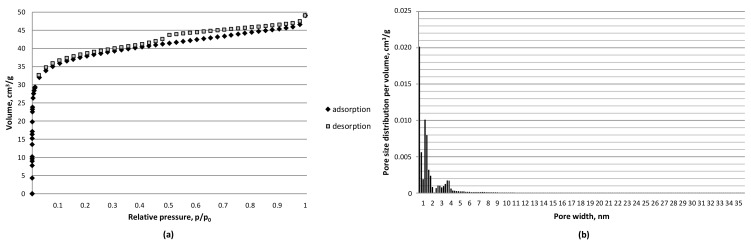
Nitrogen isotherm (−196 °C) (**a**) and pore size distribution per pore volume (**b**) for olive pomace-derived carbon materials produced at 800 °C, under carbon dioxide atmosphere and pressure of 3 MPa.

The olive pomace-derived carbon materials resulting from the carbonization process performed at 800 °C under the pressure of 0.1–3 MPa and carbon dioxide atmosphere demonstrated a micro-mesoporous structure. The exemplary nitrogen isotherm for carbon materials generated under 3 MPa presented in Figure 2a shows a relatively high uptake at the low p/p_0_ pressures characteristic of microporous materials and a narrow hysteresis loop indicative of the occurrence of a certain amount of irregular slit-like-shaped mesopores [45].

**Figure 3:**
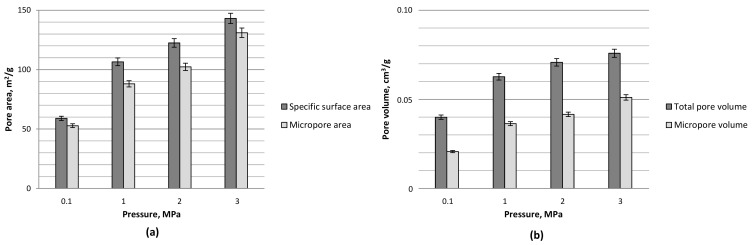
Porous structure parameters: (**a**) specific surface area and micropore area and (**b**) total pore volume and micropore volume, based on nitrogen isotherms at −196 °C for olive pomace-derived carbon materials produced at 800 °C, carbon dioxide atmosphere, and under the pressure of 0.1–3 MPa.

The specific surface area, the total pore volume, as well as the micropore volume and area determined on the basis of the nitrogen isotherm at −196°C are given in Figure 3. An increase in carbonization pressure resulted in enhanced porous structure development. The most pronounced rise (of 81%) in the specific surface area value was observed with a pressure increase from atmospheric to 1 MPa under the carbonization and activation conditions employed. Further increases in process pressure gave relative rises of 15% and 18%, respectively, at 2 and 3 MPa (see Figure 3a). A clear advantageous effect of pressure on the porous structure development of the materials tested was also reported in terms of the total pore volume. In this case, the most significant increase was also observed with a pressure rise from ambient to 1 MPa (57%), followed by 13% and 7% rises with further pressurization from 1 to 2 MPa and from 2 to 3 MPa, respectively (see Figure 3b).

The enhanced development of porous structure with increasing pressure was accompanied by a decrease in the average pore diameter value (see Table 2). While carbon materials developed under 1 and 2 MPa showed similar values of average pore diameter, a clear increase in the development of smaller pores could be seen when the values reported under atmospheric pressure, 1–2 MPa, and 3 MPa were compared. The positive effect of pressure on porous structure development may be related to the enhanced release of volatiles and moisture, as well as to a partial decomposition of the carbon structure under carbon dioxide atmosphere, high temperature, and elevated pressure. Such effects have been previously reported for biomass-derived chars [39,40], bituminous coal [36,40], and lignite [37,40] when pressure was considered as the sole agent affecting the porous structure development of carbon materials at high temperature; they were also observed for lignite chars under combined activation with pressure and carbon dioxide in the carbonization step [38].

**Figure 4:**
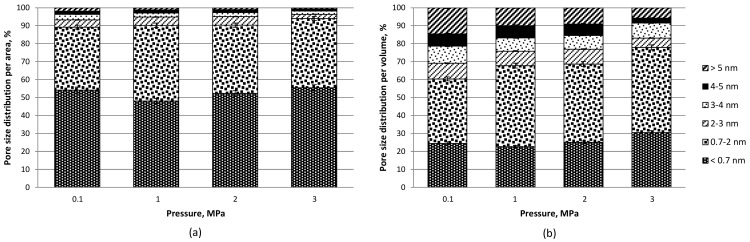
Distribution of: (**a**) pore area and (**b**) volume based on nitrogen isotherms at −196 °C and DFT method for olive pomace-derived carbon materials produced at 800 °C, under 0.1–3 MPa and with carbon dioxide activation.

The significance of volatiles content for microporosity growth has been previously reported for lignite chars developed at 1000 °C under the pressure of 1–4 MPa [37]. This observation is in good agreement with the results reported in this study, where for a parent material of high volatiles content (see Table 1), the share of microporosity in the carbonization product was over 80% when the area was considered, and over 60% when the volume was considered, with a maximum of 94% in area and 78% in volume for the carbon material developed under the maximum pressure of 3 MPa (see Figure 2b and Figure 4). It is also worth noting that the share of supermicropores (0.7–2 nm) rose with carbonization pressure in the range of 1–3 MPa from 23% to 31% per volume and from 48% to 56% per area. The amount of mesopores in carbon materials developed under the pressure of 1 and 2 MPa was about 30% (by volume) and 10% (by area) and dropped to 22% in volume) and 6% in area under the maximum pressure of 3 MPa. The application of pressure in carbon dioxide activation in the study presented proved also to be more effective in microporosity development than a combined chemical (KOH) and physical activation (H_2_O–CO_2_) of olive kernels at 800–900 °C, which resulted in 50–70%of microporosity content [17]. The considerable share of micropores, and in particular, the rise in the amount of supermicropores therefore makes carbonization pressure a parameter of interest when production of materials for various new applications, including double-layer electric capacitors, is concerned [20]. The relatively high share of ultramicropores reported for materials developed under pressurized conditions may be considered advantageous in the potential applications of carbon materials for CO_2_ capture [28].

**Figure 5:**
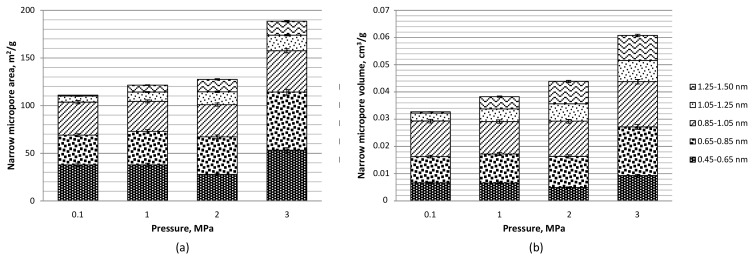
Narrow micropores: (**a**) area and (**b**) volume based on carbon dioxide isotherms at 0 °C and Monte Carlo method for olive pomace-derived carbon materials produced at 800 °C, under 0.1–3 MPa and with carbon dioxide activation.

The development of narrow microporosity (pores of a diameter of 0.45–1.5 nm) quantified with the use of carbon dioxide sorption isotherm data and the Monte Carlo method was also enhanced with pressure increase in the carbonization step. This was reflected in the respective rise in micropore area and volume (see Figure 5). The majority of pores were ultramicropores constituting approximately 50–60% of the narrow micropore area and 40–50% of the micropore volume. A characteristic feature was also the development of pores over 1.25 nm under the pressurized conditions when compared to carbon materials carbonized under atmospheric pressure. In the previous work on carbon dioxide and pressure activation of olive stones, no significant effect of pressure on microporosity development was reported, which may be related to a very narrow pressure range tested there (0.1–1 MPa) and low carbonization temperature (510 °C) [18]. Clearly, the rise in pressure to values of 2–3 MPa under the elevated temperature of 800 °C and a carbon dioxide atmosphere has a positive effect on the development of a porous structure in olive residue-derived carbon materials.

**Figure 6:**
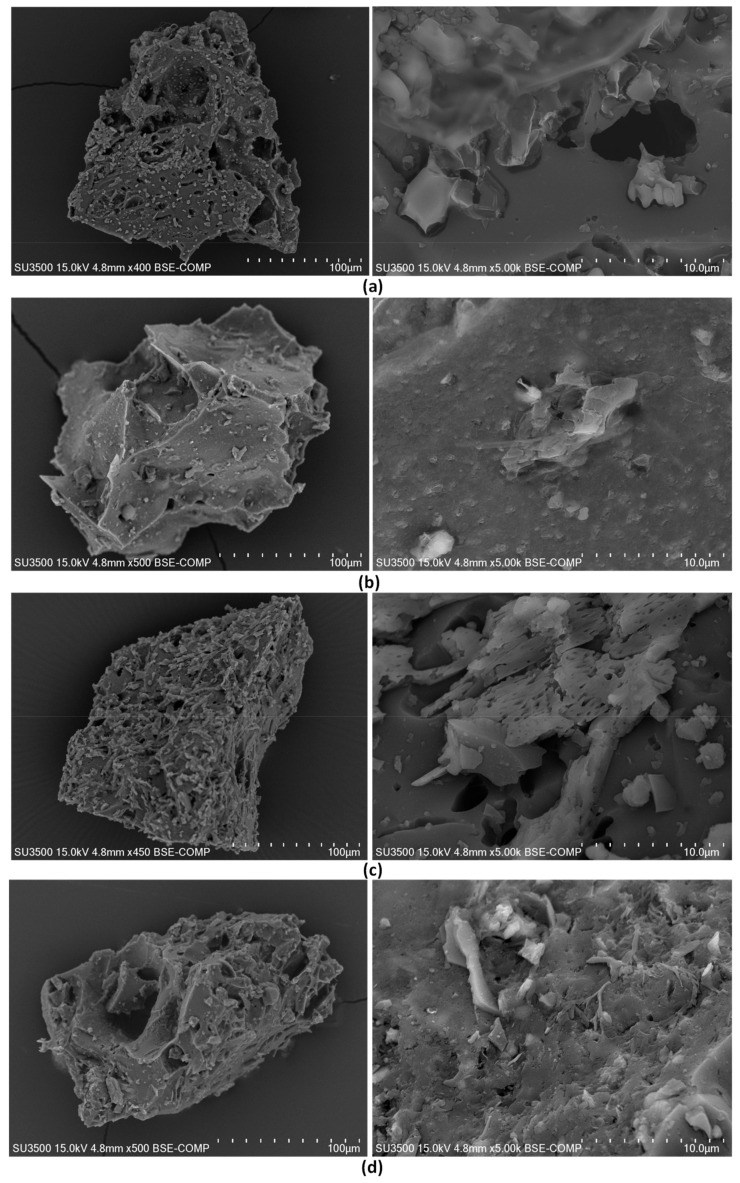
Scanning electron microscopy (SEM) images of olive pomace-derived carbon materials produced at 800 °C, under carbon dioxide atmosphere and pressure of: (**a**) 0.1 MPa, (**b**) 1 MPa, (**c**) 2 MPa, and (**d**) 3 MPa.

The texture of the resulting carbon materials was analyzed on the basis of SEM images (Figure 6). The surface of carbon materials developed under lower pressure was smooth and compact when compared to the surface of chars developed under elevated pressure (see Figure 6a,b vs Figure 6c,d, right). The cavities visible in the SEM images of materials developed under 1–2 MPa were sparse and relatively large (Figure 6a,b vs Figure 6c,d, right). The application of a higher pressure in the carbonization step resulted also in a less uniform and more complex surface structure, with a larger number of smaller fissures at the expense of larger cracks characteristic of lower applied pressures (Figure 6d, right). The higher the pressure applied, the higher the surface roughness and the more developed the porous structure observed (Figure 6a–d, left). This was the result of the enhancing effect that the higher pressure had on moisture release, devolatilization, and thermochemical conversion of the carbonaceous material under carbon dioxide atmosphere [38].

## 4. Conclusions

The following conclusions may be drawn on the basis of the experimental findings of the study performed:

1. The combination of elevated pressure, carbon dioxide atmosphere, and high carbonization temperature results in the development of microporous olive pomace-derived carbon materials.
2. The carbonization pressure may be considered a useful activation parameter when the production of materials for various new applications is considered, since it contributes to the development of supermicropores and ultramicropores under the carbonization conditions applied.
3. The combination of solely physical activation parameters, covering elevated pressure and temperature under carbon dioxide atmosphere, may be considered promising in terms of the development of effective methods of biowaste valorization, including biowaste less suitable for porous materials production.

## Figures and Tables

**Table 1:** Physical and chemical properties of olive pomace.

Parameter, Unit	Value
**Proximate analysis**	
Moisture ^1^, %w/w	5.53
Ash ^2^, % w/w	7.54
Volatiles ^3^, % w/w	70.00
Fixed carbon ^4^, % w/w	16.93
**Ultimate analysis**	
Sulfur ^5^, % w/w	0.12
Carbon ^6^, % w/w	48.56
Hydrogen ^6^, % w/w	6.76
Nitrogen ^7^, % w/w	1.13
Oxygen ^8^, % w/w	30.98
**Heating value**	
Higher heating value ^9^, kJ/kg	19,460
Lower heating value ^9^, kJ/kg	17,990

^1^ PN-EN ISO 18134-3:2015-11, ^2^ PNEN ISO 18122:2016-01, ^3^PN-G-04516:1998, ^4^PN-G-04516:1998 calculated by difference, ^5^PN-EN ISO 16994:2016-10, ^6^PN-EN ISO 16948:2015-07, ^7^PN-G-04571:1998, ^8^PN-G-04516:1998 calculated by difference, ^9^PN-EN ISO 18125:2017-07.

**Table 2:** Average pore diameter and mode diameters determined on the basis of nitrogen isotherm using the density functional theory (DFT) method and carbon dioxide isotherm using the Monte Carlo (MC) method for olive pomace-derived carbon materials produced at 800 °C, under carbon dioxide atmosphere and pressure of 0.1–3 MPa.

Pressure, MPa	Average Pore Diameter, nm N_2_ Isotherm −196 °C	Mode (DFT), nm N_2_ Isotherm −196 °C	Mode (MC), nm CO_2_ Isotherm 0 °C
0.1	2.71	0.57	0.63
1	2.35	0.60	0.55
2	2.31	0.55	0.60
3	2.12	0.57	0.55
